# Stage-Specific Tulane Virus Replication in Zebrafish: Permissive in Embryos, Restricted in Larvae

**DOI:** 10.1007/s12560-026-09708-z

**Published:** 2026-09-17

**Authors:** Sahaana Chandran, Kristen E. Gibson

**Affiliations:** Department of Food Science, Center for Food Safety, University of Arkansas Division of Agriculture, Fayetteville, AR 72704 USA

**Keywords:** Norovirus, Glycan, Cellular tropism, Surrogate

## Abstract

Human norovirus (HuNoV), the leading cause of foodborne illness in the United States and acute gastroenteritis globally, replicates in the zebrafish embryo and larvae model. However, due to challenges in obtaining well-characterized HuNoV positive stool specimens for research, demonstrating replication of Tulane virus (TuV), a surrogate of HuNoV, in the zebrafish embryo and larvae model is valuable to study HuNoV infection mechanisms and subsequent prevention and control strategies. Three nL of TuV (9 log RNA copies/mL or 7 log PFU/mL) were microinjected into either fertilized zebrafish eggs (within 4-hours post-fertilization [hpf]) or the yolk of 3-day post-fertilization larvae. Daily, 10 embryos or larvae were pooled into a single sample, with two samples collected per day up to 5 days post-infection (dpi). Viral RNA was quantified using droplet digital PCR. While TuV replicated in zebrafish embryos, with a 2-log increase by 3 dpi, no replication was observed in zebrafish larvae. Developmental regulation of sialylated glycans in zebrafish was investigated as a potential explanation for the absence of TuV replication in larvae. Zebrafish embryos express α-2,6-linked sialic acid (SA) residues, which decrease from 6 to 48 hpf, while α-2,3 sialylation increases later. Because TuV specifically binds to α-2,6-linked SA but not to α-2,3-linked SA, these developmental changes in sialylation were hypothesized to contribute to the absence of replication in larvae. To test this hypothesis, zebrafish embryos were microinjected with 3 nL of *Sambucus nigra* lectin (100 mg/mL), which binds α-2,6-linked SA, followed by TuV microinjection. However, lectin treatment did not inhibit replication of TuV, suggesting TuV may utilize other receptors in zebrafish embryos. While TuV replicates exclusively in zebrafish embryos, the previous reports of HuNoV replication in both embryonic and larval stages reveal potential limitations of TuV as a surrogate for evaluating prevention and control strategies against HuNoV transmission through food and environmental routes.

**Supplementary Information:**

The online version contains supplementary material available at https://doi.org/10.1007/s12560-026-09708-z.

## Introduction

Human norovirus (HuNoV) remains the leading cause of acute viral gastroenteritis worldwide (Carlson et al., 2024), responsible for significant morbidity and an estimated annual economic burden of 60 billion USD (Bartsch at al., 2016). This virus is also responsible for most foodborne illnesses in the United States resulting in an estimated 5.5 million cases annually (Scallan Walter et al., 2025). Despite its public health importance, progress in understanding HuNoV biology, pathogenesis, and host interactions has historically been limited due to challenges associated with cultivating the virus in laboratory systems and the lack of robust and accessible animal models (Chandran and Gibson 2024a).

While recent advances in human intestinal enteroids (HIE) and the use of zebrafish embryos and larvae have provided new avenues for studying HuNoV pathogenesis (Ettayebi et al., 2016; Van Dycke et al., 2019; Tan et al., 2023), investigation of HuNoV control strategies remains constrained by challenges in obtaining reliable and well characterized HuNoV positive stool specimens for experimental infection studies, including variability in viral load, sample quality and suitability for downstream processing. To overcome these limitations in virus availability, Tulane virus (TuV) is often used as a surrogate for HuNoV. Both TuV and HuNoV belong to the *Caliciviridae* family and share key structural characteristics including the use of similar attachment factors, such as histo-blood group antigens (HBGAs) and sialic acids (SA), to initiate infection (Farkas et al., 2008, 2010; Zhang et al., 2015). Unlike HuNoV, TuV can be propagated in vitro making it a useful model for investigating the efficacy of proposed HuNoV prevention and control strategies as well as viral attachment, entry, and replication processes that may parallel those of HuNoV. Importantly, previous work from our group demonstrated TuV replication occurs in zebrafish embryos which has also been reported for select HuNoV genotypes (Chandran & Gibson., 2024b; Tan et al., 2023). Studying TuV infection across different developmental stages of zebrafish could therefore provide valuable insights into factors influencing viral replication and host susceptibility and offer additional support for its continued use as a HuNoV surrogate.

HuNoV tropism is often determined by the presence of specific cell-surface glycans (Tenge et al., 2021). Glycan composition and expression patterns can vary between tissues and can also change during host development. In addition to their role as attachment factors, alterations in host glycan availability and processing can influence HuNoV infection outcomes, as demonstrated by recent studies showing that fucosylated glycans and microbiota-derived fucosidase activity affect HuNoV replication (Peña-Gil et al., 2025). In zebrafish, sialylated glycan expression is highly dynamic and undergoes significant shifts during early development (Hanzawa et al., 2016; Yamakawa et al., 2018). Specifically, zebrafish embryos express α−2,6-linked sialic acid (SA) residues, which decrease from 6 to 48 h post fertilization, while α−2,3 sialylation increases later. Because TuV specifically binds to α−2,6-linked SA but not to α−2,3-linked SA, these developmental changes can influence the susceptibility of zebrafish embryos and larvae to viral infection.

The objective of this study was to evaluate the replication of TuV in zebrafish larvae to further support its use as a HuNoV surrogate and to investigate whether α−2,6-linked SA residues contribute to viral infection in zebrafish model based on the shifts in glycan expression during early development. Additionally, lectin-blocking experiments were performed to assess whether masking these glycan residues affects viral replication. By comparing viral replication dynamics between developmental stages and examining potential receptor interactions, this study aims to further define the mechanisms underlying TuV infection in zebrafish and to strengthen the utility of this model for studying HuNoV pathogenesis and strategies for prevention and control.

## Materials and Methods

### Zebrafish Husbandry and Environmental Conditions

Adult wild-type zebrafish of the AB strain were obtained from the Zebrafish International Resource Center (ZIRC, Eugene, OR). The fish were maintained in a standalone tabletop eRack recirculating aquaculture system (Aquaneering, Inc., San Marcos, CA) which included carbon filters, an ultraviolet sterilizer, and a biological fluidized bed filter. The water temperature was maintained at 28 °C and followed a 14-hour light/10-hour dark cycle. To obtain embryos, adults were placed in mating cages for spawning, and the resulting fertilized eggs were kept in petri dishes filled with Danieau’s solution consisting of 1.5 mM HEPES, 17.4 mM NaCl, 0.21 mM KCl, 0.12 mM MgSO_4_, 0.18 mM Ca(NO_3_)_2_ and 0.6 µM methylene blue. All experimental procedures involving zebrafish were conducted in compliance with the guidelines of the Institutional Animal Care and Use Committee at the University of Arkansas, Fayetteville, under protocols AUP22020 and AUP23012.

### Tulane Virus Propagation and Quantification

Tulane virus was provided by Cincinnati Children’s Hospital Medical Center (Cincinnati, OH) and was propagated in LLC-MK2 monkey kidney cells (ATCC CCL-7; Manassas, VA). The production, storage, and titration of viral stocks were performed using the methods described by Arthur and Gibson (2015) with minor modifications. Following a 15 min centrifugation at 3000 × g and 4 °C, the harvested supernatant was passed through a 0.45 μm pore size bottle-top vacuum filter (Corning Inc., Corning NY). This filter was pre-treated with 1% Tween 80 and subsequently washed with 1X phosphate-buffered saline (PBS). Finally, the filtered viral supernatant was aliquoted into 1 mL cryovials and stored at −80 °C.The concentration of the TuV stock was 7 log_10_ PFU/mL or 9 log_10_ viral RNA copies/mL.

### Microinjection of TuV into Zebrafish Embryos and Larvae

For virus microinjection, fertilized zebrafish eggs were collected and rinsed three times with 0.3X Danieau’s solution. Embryos were then arranged in a Petri dish containing 1.5% agarose molded with six rows of V-shaped grooves to facilitate positioning during injection. Glass capillary needles were prepared using a micropipette puller equipped with a heat filament (Sutter Instruments, Novato, CA, USA). Microinjections were performed using an M3301R manual micromanipulator (World Precision Instruments, Sarasota, FL) coupled with a Nanoject III microinjector (Drummond Scientific, Broomall, PA). Each embryo was injected into the yolk within 6 hpf with 3 nL of Tulane virus (TuV). For larvae injections, 3 days post fertilization (dpf) larvae were anesthetized in 0.3 X Danieau’s solution supplemented with 0.4 mg/mL tricaine (Sigma-Aldrich) for 2 min. Anesthetized larvae were positioned in a 1.5% agarose mold containing six rows of angled grooves, oriented with the yolk sac facing the injection needle. Each larva received 3 nL of TuV into the yolk. Control embryos and larvae were injected with an equivalent volume (3 nL) of 1X PBS. Following microinjection, embryos and larvae were transferred to 6-well plates containing 10 mL of 0.3X Danieau’s solution per well, with ten embryos or larvae per well. Plates were maintained at 32 °C under a 14 h light/10 h dark photoperiod. Development and survival were monitored daily; deceased individuals were removed, and the medium was refreshed each day. For downstream analyses, ten embryos or larvae were pooled to constitute one biological sample. Two pooled samples were collected at each time point up to 5 days post-infection (dpi), with the 0 dpi samples collected within 1 h-post infection. Samples were placed in 2 mL tubes, and stored at − 80 °C until further processing.

### Sequential Microinjection of *Sambucus nigra* Lectin and TuV into Zebrafish Embryos

Fertilized zebrafish embryos were microinjected with 3 nL of *Sambucus nigra* lectin (SNL) (100 µg/mL in PBS) (Vector Laboratories, Newark, CA) within 2 hpf. After microinjection, the embryos were transferred to 6-well plates having Danieau’s solution. After 2 h, the embryos were microinjected again with 3 nL of TuV suspension. The embryos were then maintained in Danieau’s solution, and two samples were collected each day until 5 dpi as described above.

### RNA Extraction from Zebrafish Embryos and Larvae

Zebrafish larvae stored at − 80 °C in 2 mL tubes were homogenized after the addition of 700 µL of TRI reagent (Zymo Research, Irvine, CA) using a handheld VWR 200 Homogenizer Unit fitted with a 5 × 75 mm flat-bottom generator probe (VWR International, LLC). The homogenizer was operated at position 1, corresponding to 5000 rpm to 8000 rpm, and samples were processed for 3 cycles of 15 s with 60 s rest intervals between cycles. The homogenates were then centrifuged at 9000 × g for 5 min for clarification. RNA extraction was performed using the Direct-zol RNA Miniprep (Zymo Research, Irvine, CA) according to the manufacturer’s protocol.

### Droplet Digital PCR for Quantification of Viral RNA Copies

To detect viral RNA copies, droplet digital PCR (ddPCR) was performed using the One-Step RT-ddPCR Advanced Kit for Probes (Bio-Rad Laboratories, Hercules, CA) with 900 nM of each primer, 250 nM of the probe, and 15 nM dithiothreitol as described in Chandran and Gibson 2024b. Data were visualized and analyzed with QuantaSoft™ version 1.7.4, and thresholds distinguishing positive and negative clusters were manually set above the negative cluster.

### Statistical Analysis

Three experimental trials were performed for all experiments, and two samples were collected per trial. Viral RNA copy numbers were log_10_ transformed prior to statistical analysis. A linear model was employed for data analysis as the data met the assumptions for normality and homoscedasticity. Estimated marginal means were used to calculate treatment means and 95% confidence intervals. Statistical differences were determined by multiple pairwise comparisons at *P* = 0.05. All analyses were conducted in R Studio (R Core Team, 2022) using the *base*,* ggplot2* (Wickham, 2016), *ggpubr* (Kassambara, 2020), *emmeans* (Lenth et al., 2021), *multcomp* (Hothorn et al., 2008), and *multcompView* (Graves et al., 2019) packages.

## Results

### Microinjection of Zebrafish Embryos with TuV

**Fig. 1:**
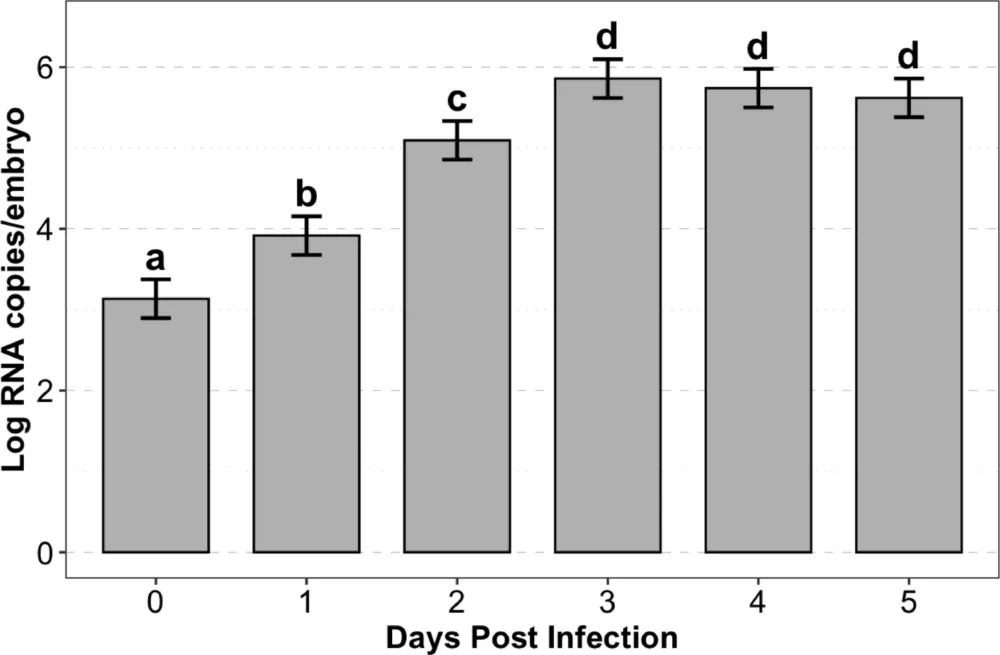
Tulane virus replication in zebrafish embryos following microinjection. Droplet digital PCR quantification of TuV RNA genome copies per embryo following microinjection from 0 to 5 dpi. Error bars represent 95% confidence intervals. Different letters indicate statistically significant differences between days (*P* ≤ 0.05) based on analysis of variance with multiple pairwise comparisons

To evaluate whether zebrafish embryos support TuV replication, fertilized eggs were microinjected with TuV and viral RNA levels were measured over a 5-day period. One-way ANOVA showed that time after infection had a significant effect on viral RNA levels (*P* < 0.001). After microinjection, TuV RNA levels increased steadily over time (Fig. 1). At 0 dpi, the estimated mean viral level was 3.13 (95% CI: 2.81, 3.46) log RNA copies per embryo. By 2 dpi, viral RNA levels increased significantly to 5.09 (95% CI: 4.76, 5.42) log RNA copies per embryo, representing nearly a 100-fold increase from the initial level (*P* < 0.001). The highest viral level was observed at 3 dpi, with an estimated mean of 5.86 (95% CI: 5.53, 6.19) log RNA copies per embryo. After this peak, viral RNA levels showed a slight decrease at 4 dpi (5.74 log copies) and 5 dpi (5.62 log copies), although these changes were not statistically significant. Despite this small decline, viral RNA levels at 5 dpi remained significantly higher than those at 0 dpi and 1 dpi (*P* < 0.05), suggesting continued viral replication and persistence of viral RNA in the embryos during development.

### Microinjection of Zebrafish Larvae with TuV

**Fig. 2:**
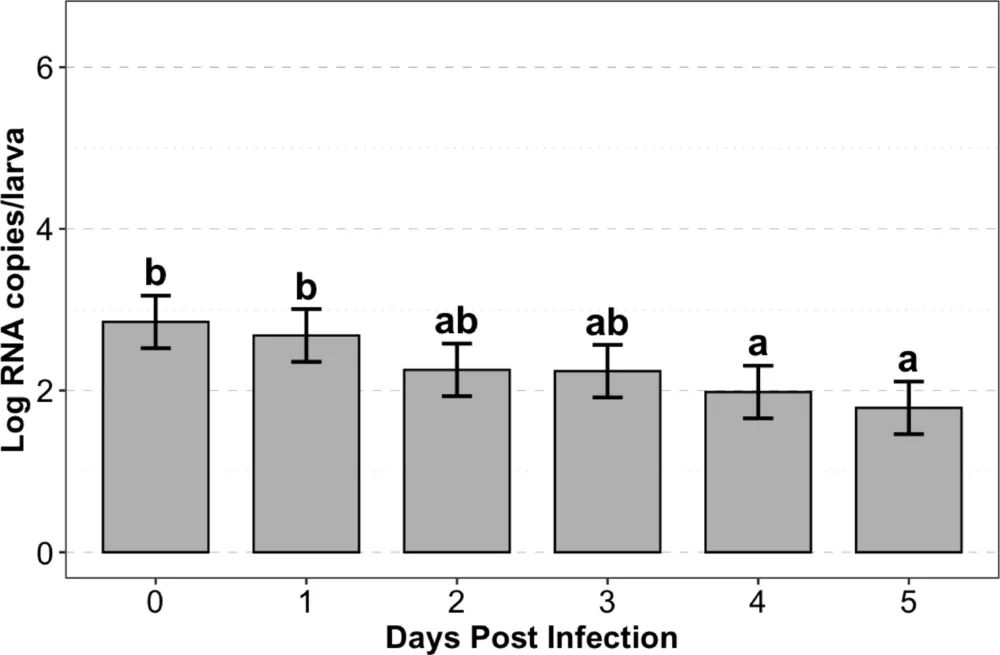
Tulane virus replication in zebrafish larvae following microinjection. Droplet digital PCR quantification of TuV RNA genome copies per larva following microinjection from 0 to 5 dpi. Error bars represent 95% confidence intervals. Different letters indicate statistically significant differences between days (*P* ≤ 0.05) based on analysis of variance with multiple pairwise comparisons

The estimated log RNA copies per larva for each day following the microinjection of 3 dpf larvae in TuV suspension are presented in Fig. 2. No replication of TuV was observed in the zebrafish larvae post-microinjection.

### Microinjection of Zebrafish Embryos with SNL

Zebrafish embryos were microinjected with 3 nL of SNL solutions at varying concentrations: 1000 µg/mL, 500 µg/mL, 200 µg/mL, and 100 µg/mL. Injection with concentrations higher than 100 µg/mL resulted in embryo lethality. Therefore, 100 µg/mL was selected for all subsequent experiments.

### Sequential Microinjection of Zebrafish Embryos with SNL and TuV

**Fig. 3:**
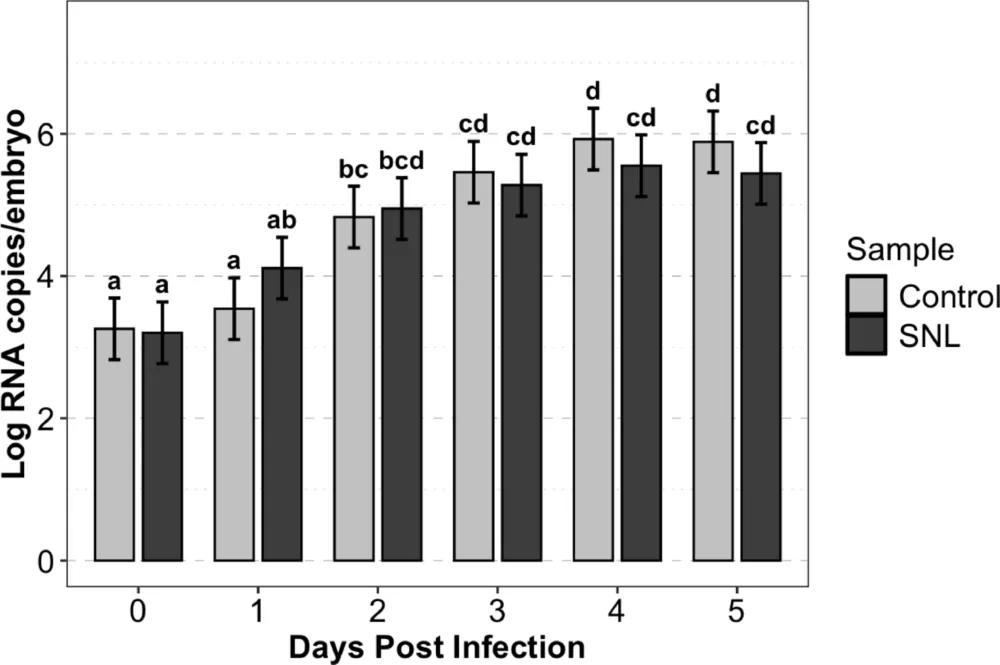
Effect of *Sambucus nigra* lectin (SNL) treatment on TuV replication in zebrafish embryos. TuV RNA genome copies per embryo were quantified by droplet digital PCR from 0 to 5 dpi following microinjection. Embryos were sequentially microinjected with SNL and TuV (SNL - dark gray bars), or with PBS followed by TuV (Control - light gray bars). Bars represent the mean log RNA copies per embryo, and error bars indicate 95% confidence intervals. Different letters above the bars denote statistically significant differences between days (*P* ≤ 0.05) based on analysis of variance with multiple pairwise comparisons

The estimated log RNA copies per embryo following sequential microinjection of SNL and TuV are shown in Fig. 3. Statistical analysis showed that the treatment (SNL vs. Control) had no significant effect (*P* = 0.627) on viral replication. There was also no significant interaction between day and treatment (*P* = 0.213), showing that the virus replicated similarly in both experimental groups. In the control group, viral RNA levels increased from 3.26 log RNA copies per embryo at Day 0 to a peak of 5.93 log RNA copies by Day 4. A similar trend was observed in the SNL treated embryos, where viral RNA levels increased from 3.20 log RNA copies at Day 0 to 5.55 log RNA copies by Day 4. At all time points between Day 0 and Day 5, no statistically significant differences (*P* > 0.05) were detected between the SNL treated and control groups.

## Discussion

In our previous work, we demonstrated that zebrafish embryos can support the replication of TuV by microinjection method (Chandran and Gibson 2024b). Building on these findings, the present study aimed to investigate whether TuV can also replicate in later developmental stages, specifically in zebrafish larvae, similar to HuNoV. In addition, this study examined whether α−2,6 linked SA residues play a role in mediating viral attachment and infection in the zebrafish embryo model.

Consistent with our earlier findings, the results of this study confirmed that zebrafish embryos support TuV replication following microinjection. In contrast, no replication was observed when TuV was microinjected into 3 dpf zebrafish larvae. This difference between embryos and larvae suggests that susceptibility to TuV infection in zebrafish may be strongly influenced by developmental stage and the availability of host factors required for viral attachment or entry.

Several strains of HuNoV have previously been shown to replicate in zebrafish larvae (Chandran et al., 2026; Tan et al., 2021, 2023; Van Dycke et al., 2019). Successful HuNoV replication in larvae has been shown to depend on the presence of terminal fucose residues that form part of HBGAs (Cuvry et al., 2022). Histo-blood group antigens are well-established attachment factors for many HuNoV strains, as demonstrated through in vitro binding assays (Huang et al., 2005; Hutson et al., 2003; Marionneau et al., 2002) as well as human volunteer challenge studies (Hutson et al., 2002; Lindesmith et al., 2003). Similarly, TuV has also been shown to interact with HBGAs in vitro (Farkas et al., 2010; Zhang et al., 2015), suggesting that glycan-mediated attachment mechanisms may play an important role in infection. Despite these similarities, the absence of TuV replication in zebrafish larvae suggests that the virus may rely on additional host factors that are not sufficiently expressed or accessible at this developmental stage.

Differences in receptor usage among caliciviruses may partly explain these observations. For example, MNV, another commonly used surrogate for HuNoV, has also been reported to fail to replicate in zebrafish larvae (Van Dycke et al., 2019). This lack of replication has been attributed to the virus’s reliance on the murine cell-surface receptors CD300lf and CD300ld for entry (Haga et al., 2016), which are not encoded in the zebrafish genome (Van Dycke et al., 2019). Although TuV does not use the same receptors as MNV, these findings illustrate how differences in host receptor availability can strongly influence replication in zebrafish models.

In addition to HBGAs, SA containing glycans have also been implicated in HuNoV attachment. For example, HuNoV has been reported to bind to SA-containing glycosphingolipids (Han et al., 2014). Similarly, TuV has been shown to bind to glycans containing α−2,6 linked SA, such as 6′-sialyllacNAc, but not to glycans containing N-glycolylneuraminic acid or α−2,3 linked SA structures (Tan et al., 2015). These findings suggest that α−2,6 linked SA residues may contribute to TuV attachment. The relevance of these glycan interactions in zebrafish may be influenced by developmental changes in glycosylation patterns. In adult zebrafish tissues including the liver, intestine, brain, ovaries, and testis α−2,3, linked SA residues are the predominant form (Yamakawa et al., 2018). In contrast, early zebrafish embryos predominantly express α−2,6 substituted sialylated N-glycans (Gabor et al., 2014). However, α−2,6 sialylation decreases during embryonic development between 6- and 48-hpf, whereas α−2,3 sialylation increases during later developmental stages (Hanzawa et al., 2016). These developmental changes in glycan composition may influence viral attachment and infection. The relatively higher abundance of α−2,6 linked SA residues during early embryonic stages could potentially facilitate TuV attachment and replication, whereas the reduced presence of these glycans in later stages may contribute to the lack of TuV replication observed in larvae.

To further investigate the role of α−2,6 linked SA in TuV infection, SNL which specifically binds to α−2,6 linked SA residues, was used to block these glycans prior to viral infection. Experimental evidence supporting the role of α−2,6 linked SA residues in TuV infection has also been demonstrated in vitro. In a previous study, SNL was used to block potential viral attachment sites on LLC-MK2 cells prior to infection (Tan et al., 2015). In that experiment, cells were treated with SNL for 60 min to allow the lectin to bind to α−2,6 linked SA residues on the cell surface. After the incubation period, the lectin was removed and TuV was added to the cells, followed by plaque assay analysis to measure viral infection. The results showed that TuV infection was reduced by approximately 32% compared to untreated cells (Tan et al., 2015). However, in the present study, sequential microinjection of SNL followed by TuV did not result in a significant reduction in viral replication compared to the control group. This observation suggests that α−2,6 linked SA residues alone may not be essential for TuV infection in zebrafish embryos, and that other host glycans or receptors may also facilitate viral entry.

In addition to developmental changes in host glycan expression, differences in the microbial environment between embryos and larvae may also influence TuV susceptibility. The zebrafish microbiome undergoes substantial developmental changes, with embryos developing in a relatively low-microbial environment and distinct microbial communities becoming established during larval development (Stephens et al., 2016). Therefore, the microbial environment encountered by 3 dpf larvae may differ considerably from that of early embryos and could represent an additional factor influencing virus infectivity as has been demonstrated with HuNoV in the HIE and zebrafish model systems (Peña-Gil et al., 2025; Santiso-Bellon et al., 2025; Yang et al., 2025; Chandran, 2026).

Another possible explanation for the lack of TuV replication in zebrafish larvae might be the developmental maturation of the innate immune system. In zebrafish, innate immune defenses begin to develop around 1 dpf, whereas embryos at 0 dpf lack a fully functional innate immune response (Liao et al., 2021). This immature immune state may permit viral replication before antiviral sensing mechanisms are established. In contrast, larvae possess a more mature innate immune system capable of mounting interferon-mediated antiviral responses and inducing interferon-stimulated genes (Tan et al., 2026), which may restrict TuV replication. However, larvae are still permissive to HuNoV infection which suggests that the zebrafish immune response is not solely responsible for the lack of TuV replication.

One limitation of the present study is the concentration of SNL that could be used in zebrafish embryos. Higher concentrations of SNL resulted in embryo lethality, and therefore a concentration of 100 µg/mL was selected. Although this concentration allowed embryo survival, it may not have been sufficient to block all available α−2,6 linked SA binding sites on host glycans. As a result, some viral attachment sites may have remained accessible, allowing TuV to bind and replicate despite lectin treatment. This limitation may partly explain why no significant reduction in viral replication was observed following SNL treatment. In addition, the distribution of SNL following yolk microinjection was not directly visualized in this study. Therefore, it remains unclear whether the injected lectin was able to efficiently translocate from the yolk to embryonic tissues and access α−2,6-linked SA residues on embryonic cell surfaces. Previous studies have demonstrated that lectins can interact with and exert biological effects in zebrafish embryos, supporting the feasibility of lectin-mediated modulation of embryonic glycans (Wang et al., 2019). In addition, recombinant lectins have been successfully delivered by microinjection in zebrafish embryos and retained biological activity (Yang et al., 2014). Future experiments using fluorescently labeled SNL would be required to confirm SNL localization and its ability to modify embryonic cell surface glycans.

Another consideration is that TuV was microinjected into the yolk of zebrafish embryos in the present study. Recent studies have demonstrated that HuNoV microinjected into the embryo yolk can disseminate to multiple embryonic cell types in zebrafish (Tan et al., 2026). Furthermore, HuNoV recovered from infected embryos can be serially passaged, demonstrating that zebrafish embryos can support the production of infectious virus (Tan et al., 2023). The increase in TuV RNA levels observed in the present study is consistent with viral replication, though we did not directly determine whether infectious virus particles were produced. Similar experiments examining TuV localization and infectivity will be important to determine viral dissemination and tissue tropism in the zebrafish embryo model.

Taken together, the results of this study demonstrate that zebrafish embryos support TuV replication, whereas zebrafish larvae do not appear to support TuV infection. The inability of TuV to replicate in larvae, despite the reported replication of several HuNoV strains in the same model, highlights important differences in virus–host interactions among caliciviruses. These differences may be driven by variations in receptor usage, glycan binding specificity, or developmental changes in host glycan expression. Furthermore, the results of the lectin-blocking experiments suggest that α−2,6 linked SA residues alone are not sufficient to explain TuV infection in zebrafish embryos, indicating that multiple host factors may contribute to viral attachment and replication. Further studies examining glycan expression patterns and receptor availability across zebrafish developmental stages will be important for clarifying the mechanisms underlying TuV infection in this model and the future suitability of TuV as a surrogate for HuNoV during evaluation of prevention and control strategies relevant to food and environmental transmission.

## Supplementary Information

Below is the link to the electronic supplementary material.

Supplementary Material 1

Supplementary Material 2

Supplementary Material 3

## Data Availability

All data supporting the findings of this study are available within the paper and its Supplementary Information files.
